# Nanoparticle Synthesis by Precursor Irradiation with Low-Energy Electrons

**DOI:** 10.1021/acsanm.4c06033

**Published:** 2025-03-06

**Authors:** Kristina Weinel, Marc Benjamin Hahn, Axel Lubk, Wen Feng, Ignacio Guillermo Gonzalez Martinez, Bernd Büchner, Leonardo Agudo Jácome

**Affiliations:** † Federal Institute for Material Research and Testing, Unter den Eichen 87, 12205 Berlin, Germany; ‡ Leibniz Institute for Solid State and Materials Research Dresden e.V., Helmholtzstr. 20, 01069 Dresden, Germany; § Institute for Solid State and Materials Physics, Technische Universität Dresden, Haeckelstr. 3, 01069 Dresden, Germany; ∥ University of Potsdam, Karl-Liebknecht-Str. 24-25, 14476 Potsdam, Germany

**Keywords:** Gold nanoparticle synthesis, Scanning electron microscopy, Heat transfer, Thermodynamic modeling, Precursor microparticle irradiation, Monte Carlo scattering simulation

## Abstract

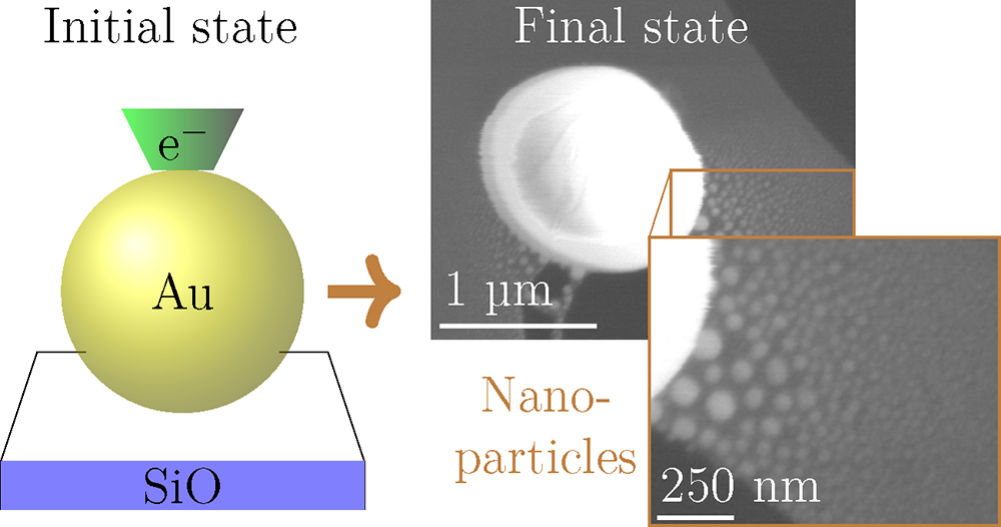

Nanoparticles (NPs) and their fabrication routes are intensely studied for their wide range of application in optics, chemistry, and medicine. Γ-ray and ion irradiation of precursor matter are established methods that facilitate tailored NP synthesis without complicated chemistry. Here, we develop and explore NP synthesis based on irradiating precursor microparticles with low-energy electron beams. We specifically demonstrate the fabrication of plasmonic gold nanoparticles of sizes between 3 and 350 nm on an amorphous SiO_*x*_ substrate using a 30 kV electron beam. By detailed comparison with electron scattering simulations and thermodynamic modeling, we reveal the dominant role of inelastic electron–matter interaction and subsequent localized heating for the observed vaporization of the precursor gold microparticles. This general principle suggests the suitability of electron-beam irradiation for synthesizing NPs of a wide class of materials.

## 1 Introduction

Due to their unique optical, electronic, and chemical properties, nanoparticles (NPs) of dimensions below 100 nm have garnered significant attention across multiple scientific domains.^1−3^ Recent advancements have expanded their applications, e.g., for plasmonics, photocatalysts, targeted drug delivery, wound healing, etc.^4−7^ NPs can be fabricated by various mechanical, physical and chemical methods,^1,8^ including the widely employed condensation from gas or plasma phases, wet chemistry and ion implantation methods.^9−12^ In comparison, γ–ray and ion irradiation techniques that synthesize NPs by decomposing a precursor material through radiolysis or thermodynamic processes,^13,14^ are relatively simple in their setup and required chemicals, while allowing control of particle sizes via radiation dose and precursor concentration.^15^

Electron beam irradiation, while being extensively used in nanotechnology and manufacturing applications, such as e-beam lithography,^16,17^ e-beam welding,^18,19^ e-beam-induced deposition,^20,21^ and additive manufacturing,^22,23^ have received only little attention in this context. While several studies demonstrated the general possibility to decompose microparticle (MP) precursors into NPs using high-energy electrons (between 80 and 300 keV) in transmission electron microscopes (TEMs),^24−26^ the feasibility at medium and low electron energies (up to 30 keV),^25,27^ which are important for cost efficient large scale NP synthesis, remains largely unexplored. Moreover, these previous studies could not provide a comprehensive understanding of the e-beam-induced synthesis process, disentangling the role of e-beam-induced heating and charging induced by complex scattering, heat, and charge diffusion processes.

**Figure 1:**
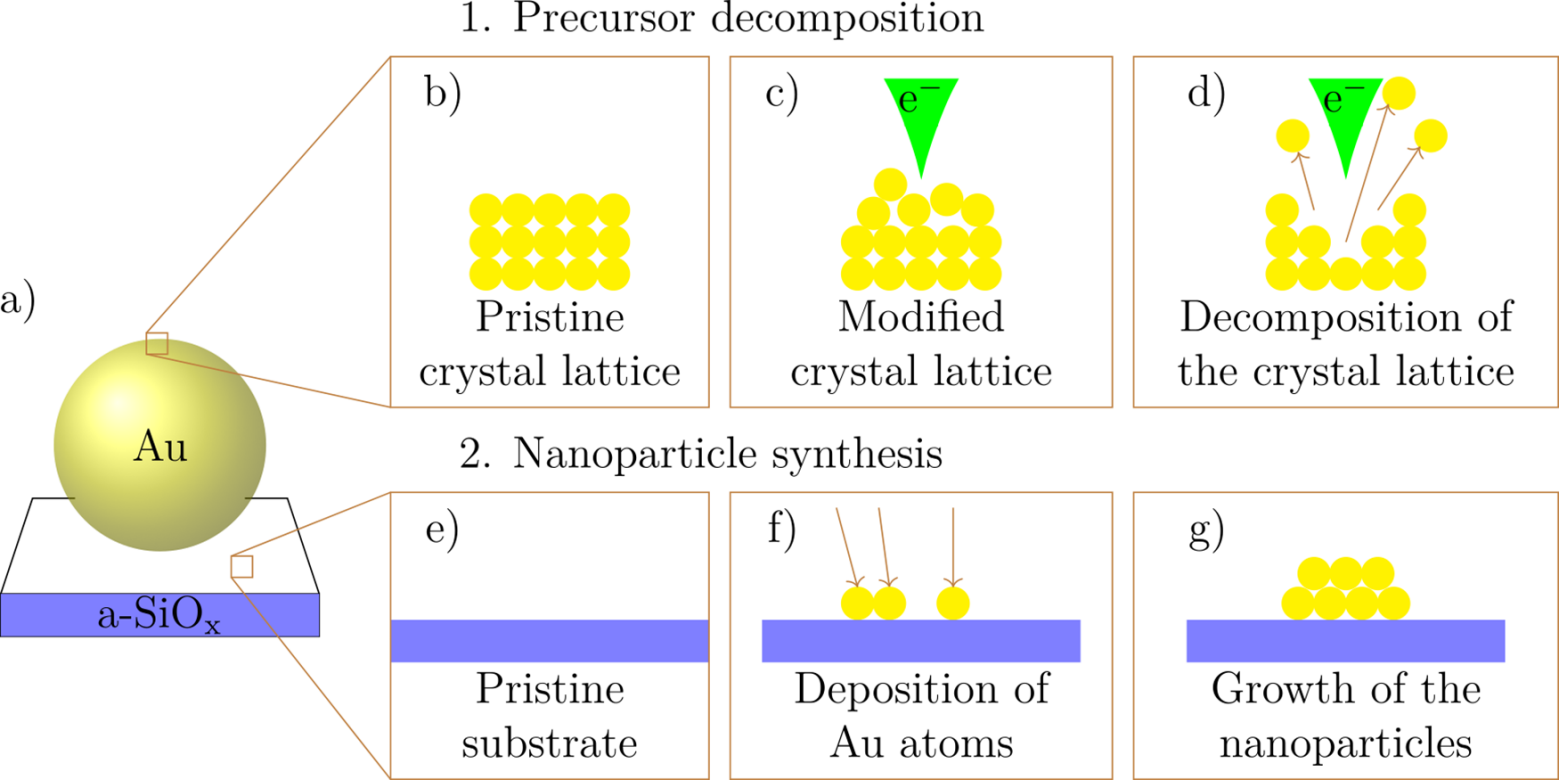
Schematics of the e-beam induced NP synthesis method: (a) Side view of the gold MP precursor (yellow) located on a thin a-SiO_*x*_ substrate (purple). (b) Gold atoms (yellow) arranged in a crystal lattice. (c) e-beam modification of the lattice by inelastic interaction. (d) Ejection of gold atoms from the MP precursor. (e) Empty pristine substrate, (f) deposition of gold atoms, and (g) growth of NPs.

To fill this gap, we study e-beam induced synthesis of NPs at low and medium electron energies in scanning electron microscopes (SEMs). The working principle of electron-beam-irradiation induced synthesis is exemplarily shown using gold MPs deposited on amorphous silicon oxide (a-SiO_*x*_) substrate as a benchmark precursor system (see Figure 1). The synthesis process can be viewed as a two-step process: First, a structural decomposition of the MP by the election beam (Figure 1b–d), followed by a subsequent formation of the gold NPs on the substrate in a second state (Figure 1e–g).

We demonstrate that Au NPs with a wide size distribution ranging from *d* = 3 nm to *d* = 350 nm can be synthesized via electron beam irradiation of the precursor MPs (see Section 2.2). The SEM’s optical degrees of freedom, such a beam energy, beam current and spatiotemporal modulation of the latter, are exploited to comprehensively probe the e-beam irradiation parameter space. Among others, this parameter study reveals a systematic dependency of the NP synthesis on the electron fluence and dose (see Section 2.2). By simultaneously detecting the backscattered and secondary electrons in the SEM the whole process is monitored in situ, thereby providing an untarnished view of the synthesis products without further sample modification or preparation (see Section 2.3). By comparing and rationalizing the findings with the help of electron-matter scattering simulations^28−30^ (see Section 2.4.1), including a cascade of inelastic scattering as well as heat (see Section 2.4.2) and charge diffusion processes (see Section 2.4.3), we provide a comprehensive understanding of the e-beam-induced matter modification.

## 2 Experimental Section

### 2.1 E-beam Irradiation Protocol

To prepare the precursor on the substrate, the following procedure was applied to spherical gold MPs (Alpha Aesar, Thermo Fisher Scientific, nominal diameter *d* = 0.8–1.5 μm). The MPs were dry-sprinkled onto a copper grid (200 mesh) coated with a holey amorphous mixture of SiO and SiO_2_ (SPI Supplies), which will be referred to as “a-SiO_*x*_” from now on. The mean thickness of the substrate, *t* = (52 ± 16) nm was measured by SEM as well as by Electron Energy Loss Spectroscopy in the TEM.

The electron beam irradiation protocol for electron-beam-induced material modification was implemented in a Quanta 3D FEG 200/600 (FEI Company) SEM. The secondary electron (SE) images, referred to as “frames” in the article when they are use for the irradiation protocol, were acquired with a built-in Everhart Thornley (ET) Detector at room temperature and pressure of *P* ≈ 3× 10^–4^ Pa. The gray values in these SE-images are indicative of the secondary electrons emitted from the specimen. Thus, dark areas, signifying the absence of secondary electrons, represent holes, whereas bright areas indicate a high amount of secondary electrons and correspond to metallic material. To produce a precise beam current control and a high beam current stability, the analytical mode of the SEM was employed, which limits the beam current to the range of discrete values between *I* = 5 nA and *I* = 48 nA. These beam currents have been calibrated by using a Faraday cup.

**Figure 2:**
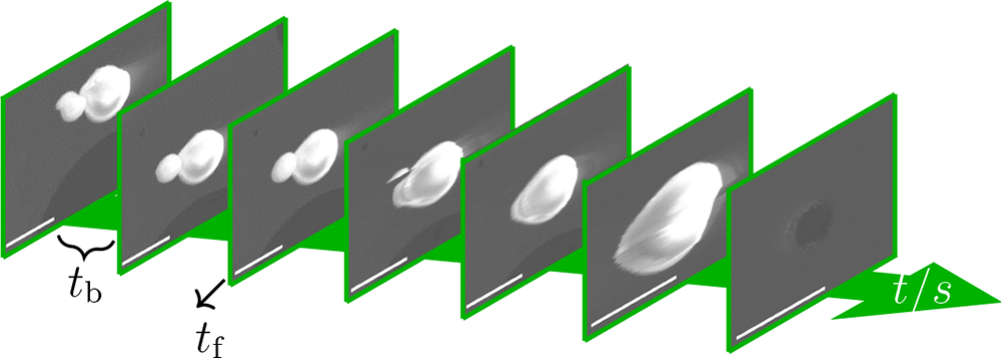
Precursor irradiation process resolved in time. In this example, an e-beam with *V* = 30 kV and *I* = 11 nA was used. Particle aggregation occurred after 5 frames and further irradiation of the MP leads to nanoparticle synthesis in the vicinity of the precursor MP. Finally, the MP is ejected, providing the view of a hole in the substrate. The scale bars correspond to 2 μm, *t*_b_ > 15 s is the time where the beam is blanked, and *t*_f_ < 1 s is the frame recording time.

Figure 2 shows a series of frames that are recorded at constant acceleration voltage, current, and working distance. In this example, *V* = 30 kV and *I* = 11 nA were used. The scan time for each frame is *t*_f_ < 1 s, whereas the blank time of the beam is with *t*_b_ > 15 s at least 1 order of magnitude longer than the scan time. The magnification may change from frame to frame, corresponding to a change of the scan field, in order to increase the current density and hence activate modification processes. We note that the number of frames considerably varied mainly due to differences in the number of frames required to observe a final MP state.

By repeatedly scanning the beam over the gold MP, different types of MP modifications, referred to as “outcomes” in the following, can be observed as shown in Figure 2. Details on the outcomes will be given in Section 2.2. These irradiation experiments started at low e-beam current. If no visible MP transformation could be observed, a second series of frames with a higher e-beam current was recorded to eventually induce a modification. In total, more than 60 gold MPs on a-SiO_*x*_ substrate were irradiated following this protocol. The obtained MP modifications indicate a stable and repeatable behavior of gold MPs on a-SiO_*x*_ substrate under the applied e-beam conditions. They, however, also exhibit a statistical scatter, likely hinting at a non negligible impact of experimental parameters other then the e-beam irradiation protocol, such as the substrate-MP contact area, which are not-well-defined.

### 2.2 E-beam Irradiation Results

**Figure 3:**
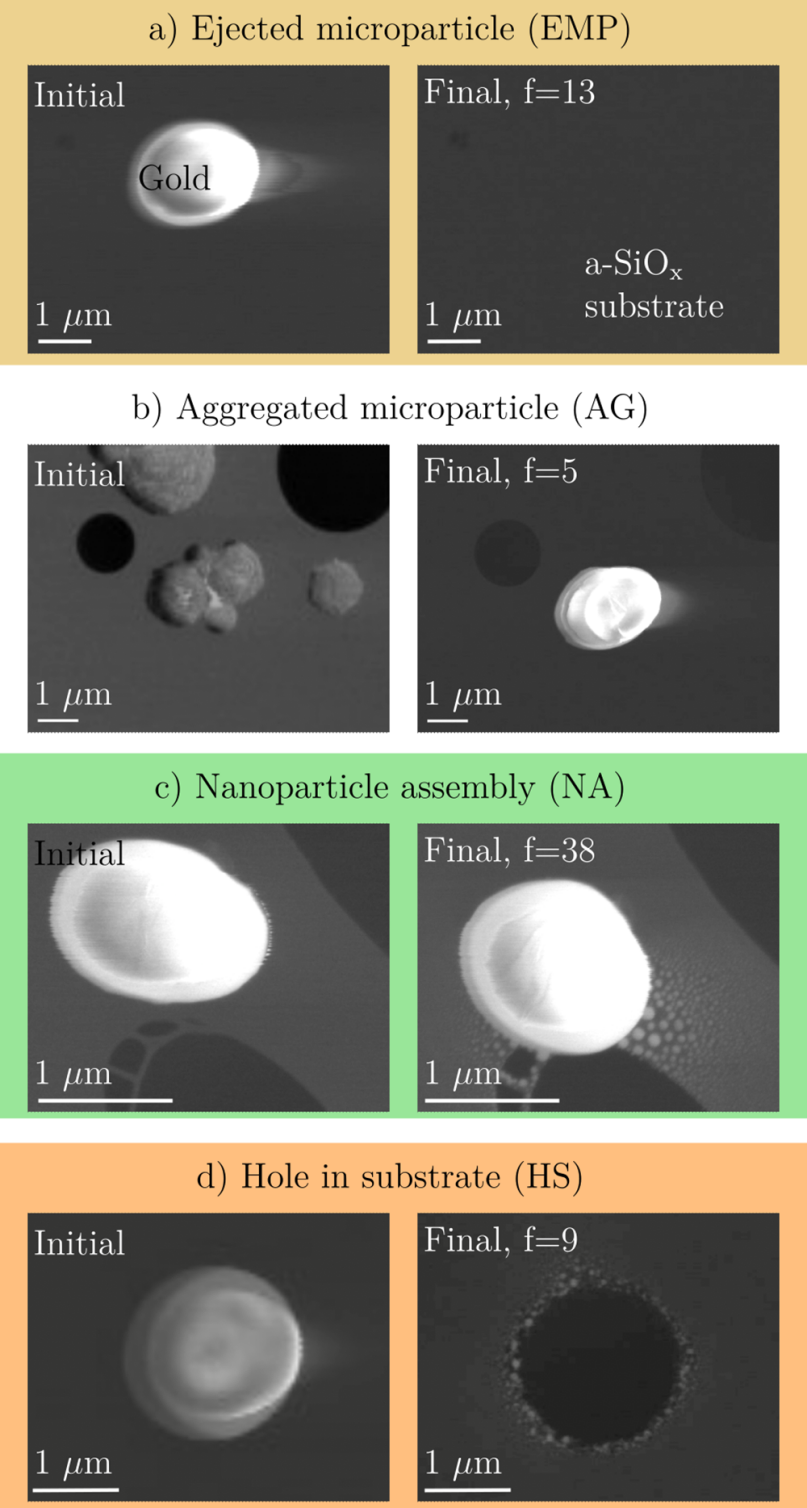
Four not necessarily exclusive outcomes: (a) ejection of MPs (EMP), (b) aggregation of MPs (AG), (c) formation of a nanoparticle assembly (NA), and (d) formation of a hole in the substrate (HS).

The final state of the MPs and the surroundings were evaluated and categorized to identify the possible outcomes after e-beam irradiation. We note that the SE-SEM images recorded after applying the irradiation protocol utilized an e-beam with *V* = 5 kV in order to prevent further modifications. In total, four different outcomes have been distinguished, as shown in Figure 3.

- Ejected microparticle (EMP) (Figure 3a): Gold MPs have been ejected from the substrate position due to the irradiation. The ejection occurs faster than the scan time and can be attributed to the charging of the MP and the substrate with the same sign and, therefore, repelling each other.
- Aggregation (AG) (Figure 3b): If multiple precursor MPs, which are in contact with each other, are irradiated, they tend to fuse to form a new single gold MP. This type of outcome is typically observed prior to other modifications and is in accordance with previous Au MP irradiation experiments.^27,31^
- Nanoparticle assembly (NA) (Figure 3c): The NA was found in the surroundings of the gold MP, while the precursor MP size was reduced.^27,31^ At higher applied currents, the NA synthesis starts already during the first frame. The size of the NPs are in the range 3 nm ≤ *d* ≤ 350 nm depending on their distance to the precursor material, whereby the larger particles form closer to the precursor MP.
- Hole in the substrate (HS) (Figure 3d): At the position of the precursor MP, a hole appeared in the thin a-SiO_*x*_ substrate (black area in the SE image). In contrast to the smooth edges of the initial holes in the substrate (see Figure 3b,c), the fabricated holes exhibit a rough edge as shown in Figure 3d. Interestingly, hole formation is always accompanied by the presence of NA. This type of outcome is attributed to severe substrate heating.

**Figure 4:**
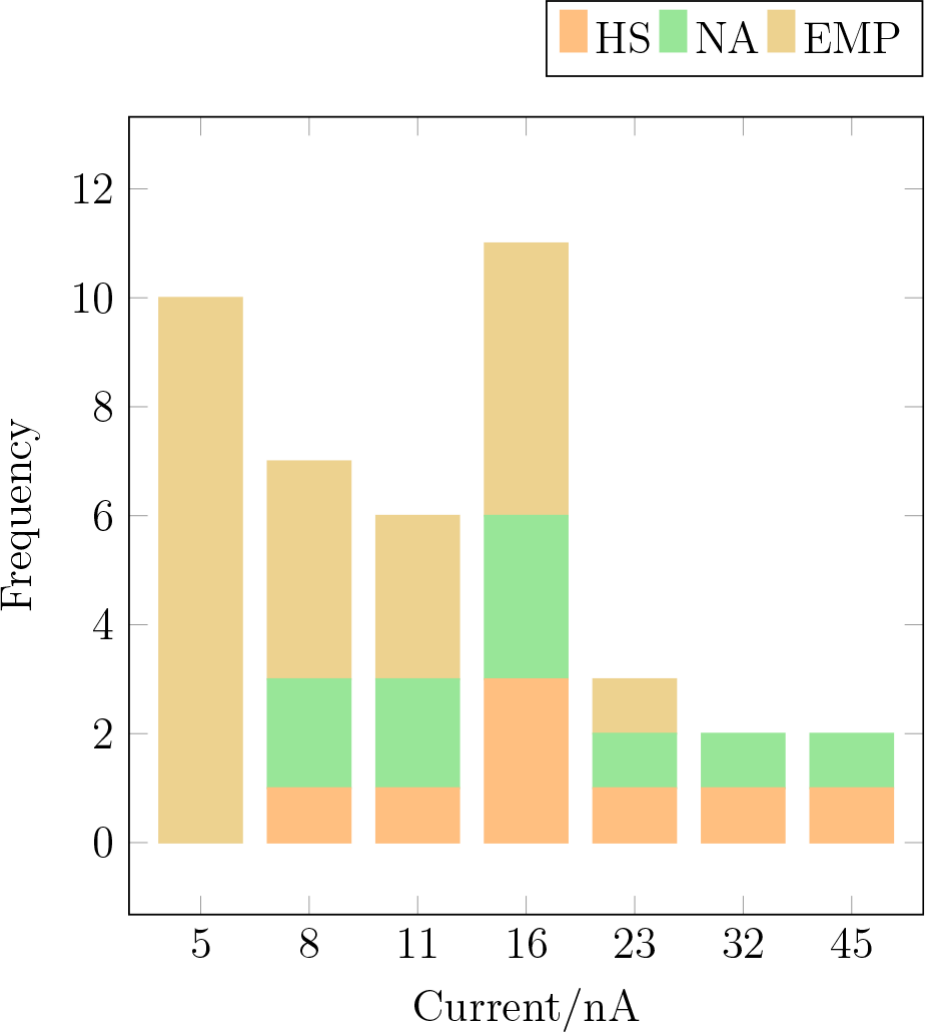
Relative frequency of the outcomes HS, NA, and EMP upon variation of the current of the applied e-beam at a constant acceleration voltage of *V* = 30 kV.

To investigate, how the e-beam current influences the type and frequency of the outcomes, the acceleration voltage and the scan time are kept constant, while the applied current changes in the range between *I* = 5 nA and *I* = 45 nA. The result is shown in Figure 4 for three observed outcomes HS, NA and EMP. AG is not observed here, because these experiments started with a single precursor MP.

At a low current of *I* = 5 nA, only EMP can be observed, which hints to charging effects that are insufficiently compensated, e.g., by electron currents through the substrate. The frequency of EMP reduces by increasing the current, and the outcomes NA as well as HS become more frequent. At a threshold current of *I* = 8 nA, the synthesis of gold NP (NA) as well as HS can be observed, which reach their maximum frequency at *I* = 16 nA. A smaller total number of results are observed at higher currents because typically the final state of the MP modification was reached at lower currents already.

The occurrence of the aggregation of multiple precursor gold MPs can be considered as a preparation step as long as it takes place as the first modification. The newly aggregated single MP counts then as one precursor particle for further treatment of the MP with an e-beam. Hence, Table 1 shows the relative frequency of AG regarding the applied current of the e-beam.

**Table 1:** Relative Frequency of AG versus Current of the E-beam

current/nA	relative frequency/%
5	0
8	30
11	40
16	20
23	10
32	0
45	0

At a low current of *I* = 5 nA, no AG was observed, implying that indeed only EMP (and no visible other modifications) occur. In the range between *I* = 8 nA and *I* = 23 nA, AG takes place with different frequencies.

All these observations indicate that there is a correlation between the kind of outcome and the applied current of the e-beam,^32^ although several unknown and uncontrollable parameters (such as contact area of MP and sample) lead to a considerable scatter.

### 2.3 Electron-Beam-Induced Charging

**Figure 5:**
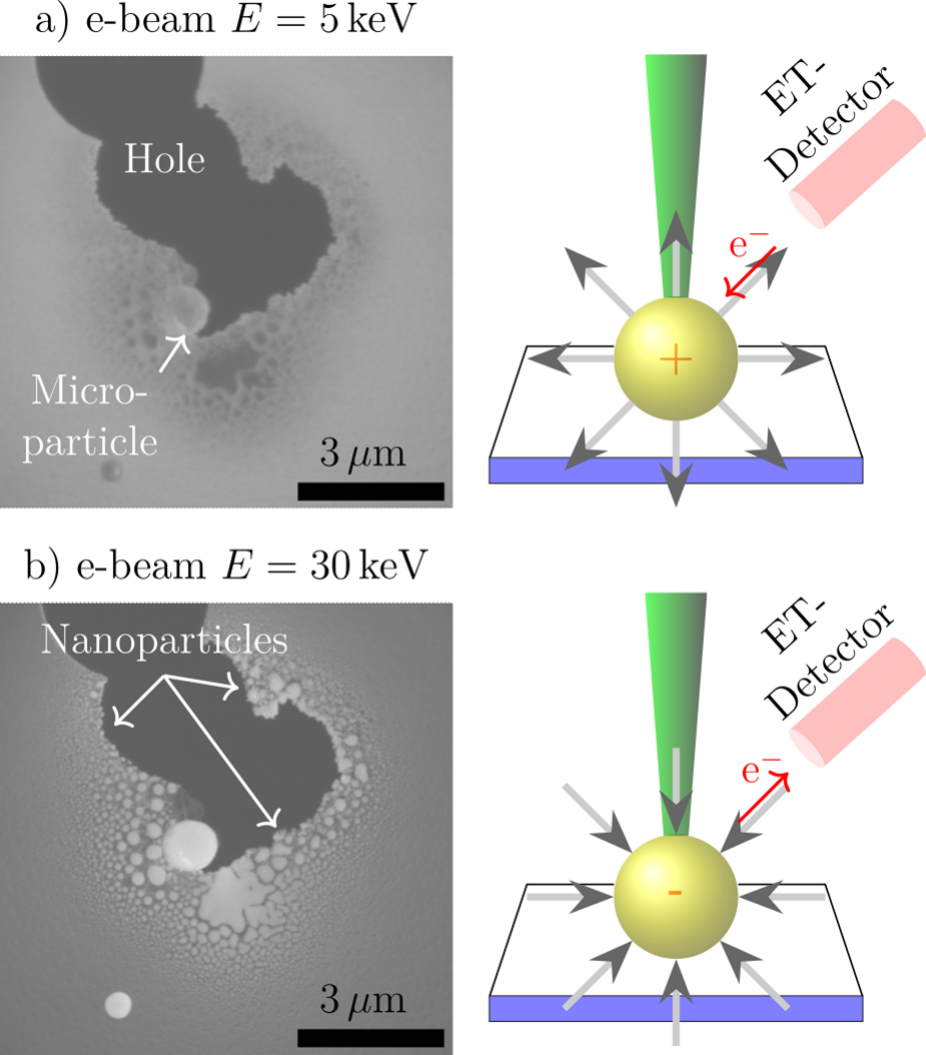
Comparison of SE images of the same sample location, acquired at different e-beam energies. (a) *E* = 5 keV and (b) *E* = 30 keV exhibit different contrasts, which are related to the number of detected SEs in the ET detector at each scan point. This signal depends not only on the SE yield of the sample but also on the path of the electrons to the detector, which differs under the influence of different additional induced electrostatic fields due to charging as sketched on the right-hand side, respectively.

To complete the experimental characterization of the irradiation, we also study the charging of the precursor MP within the electron beam. We assess the charging from SE images acquired at the same location, but with different incident energies as exemplarily shown in Figure 5.

The left image in Figure 5a shows an SE image recorded with an e-beam of *E* = 5 keV. The dark area is a hole in the a-SiO_*x*_ substrate while an assembly of NPs is in its surroundings. The residual MP is denoted by the white arrow. The image on the right-hand side represents a sketch of the e-beam (green) impinging a precursor MP (yellow), which is located in a thin substrate (blue-white). The ET-detector (light red) is situated adjacent to the pole piece within the SEM chamber. As depicted in the sketch, a positively charged MP is represented by electrostatic field lines (gray), which emanate outward and facilitate the acceleration of an electron toward the MP (red arrow).

An SE image recorded with an e-beam of *E* = 30 keV is displayed on the left in Figure 5b. The NA surrounding the hole is visible as small bright spots. On the right-hand side, a negative charged MP by is depicted by electrostatic field lines pointing toward it, facilitating electron acceleration outward (red arrow).

**Figure 6:**
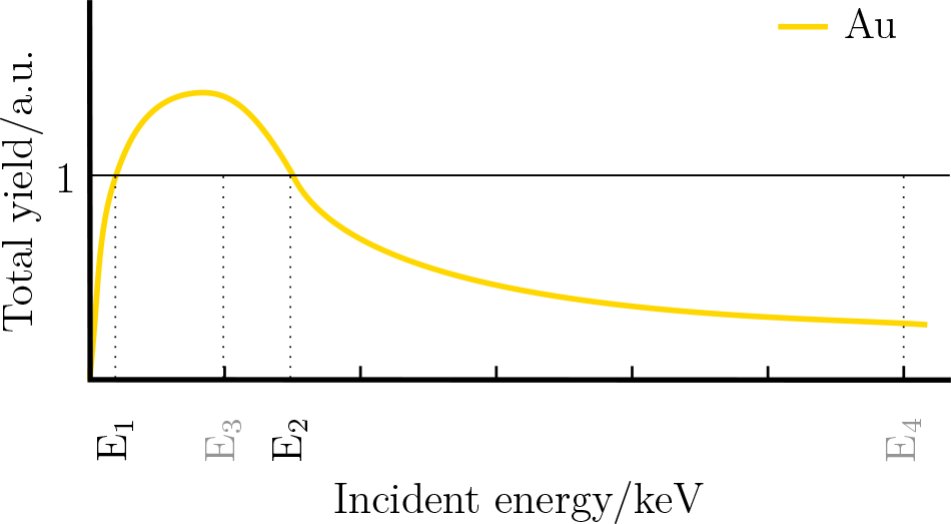
Sketch of the total yield depending on the incident electron energy for gold (yellow). *E*_1_ are several eV, *E*_2_ = 7.5 keV for gold. Regimes above a total yield of 1 are positively charged, and regimes below 1 are negatively charged. *E*_3_ = 5 keV corresponds to the e-beam energy used for these charging experiments. *E*_4_ = 30 keV corresponds to the applied energy in the fabrication process.

Positive and negative charging can occur in an insulated sample depending on the incident electron energy and material properties, such as shape, density and atomic species.^33^Figure 6 shows an example of the total yield versus the incident energy of gold. The total yield are the sum of the emitted SEs and BSEs from the specimen.

The number of emitted electrons and the number of incident primary electrons (PE) can compensate each other, leading to a total emission yield of 1 where no charging occurs (see *E*_1_ and *E*_2_ in Figure 6). Between *E*_1_ and *E*_2_, positive charging takes place and for the other regimes of incident energies, negative charging occurs.^34,35^ Typical values in buld Au are for *E*_1_ several eV and for *E*_2_ ≈ 7.5 keV.^36^

Combining both the gray values of the SE image in Figure 5a, which exhibits a relatively low contrast, and the information from Figure 6 leads to the conclusion that the MP is positively charged. Hence, the arising electrostatic field, with field lines pointing outward, influences in turn the emitted electrons. Particularly, the emitted electrons are decelerated by the field and attracted back to the MP. Therefore, less electrons reach the ET-detector and the contrast is low.

Figure 5b represents an SE image recorded at an incident beam energy of *E*_4_ = 30 keV which is the applied energy used in the irradiation protocol. A relatively high contrast is shown and again combining this information together with those from Figure 6 leads to the conclusion that the gold MP is negatively charged. Hence, the arising electrostatic field, with field lines pointing in the direction of the MP, influences the emitted electrons in a way that they are accelerated toward the ET-detector, which increases the contrast in the image.

### 2.4 Scattering and Thermodynamic Simulations

Interaction between the e-beam and matter includes a multitude of physical processes, which can be classified as elastic and inelastic scattering, where the cross-section of each interaction depends mainly on the e-beam energy and the type of matter constituents.^33^

To address the underlying physical mechanisms driving the fabrication of NPs, two numerical simulations were carried out. First, we used Monte Carlo scattering simulations of electrons impinging on a spherical gold MP standing on a SiO substrate to determine the sign of the charging as well as the magnitude of the deposited energy. Second, the latter was used to estimate the temperature increase of the MP via thermodynamic modeling.

#### 2.4.1 Monte Carlo Scattering Simulations

For the scattering simulations, we utilized the *Geant4* 10.06.p03 framework and the *TopasMC* 3.7 interface, employing the *Livermore* scattering models as well as the “*g4em-standard_SS*” physics list with deactivated multiple-scattering processes.^29,37^ The following additional processes were activated: Fluorescence, Auger, AugerCascade, and PIXE, thereby taking into account the majority of inelastic scattering processes at low beam energies with the notable exception of (volume) plasmon excitation. The latter results in an underestimation of the stopping power. The energy loss due to bulk plasmons is, however, low compared to other losses (notably core electron excitation), rendering the total contribution to the energy deposited in the precursor MP small in spite of their large scattering cross sections.

The gold and SiO have a density of  and , respectively. A detailed discussion of the particle scattering simulation of microscopic and nanoscopic gold structures can be found in the work of Zutta and Hahn.^38^ For evaluating the locally deposited energy, the “parallel-world” feature of *Geant4* was used.

**Figure 7:**
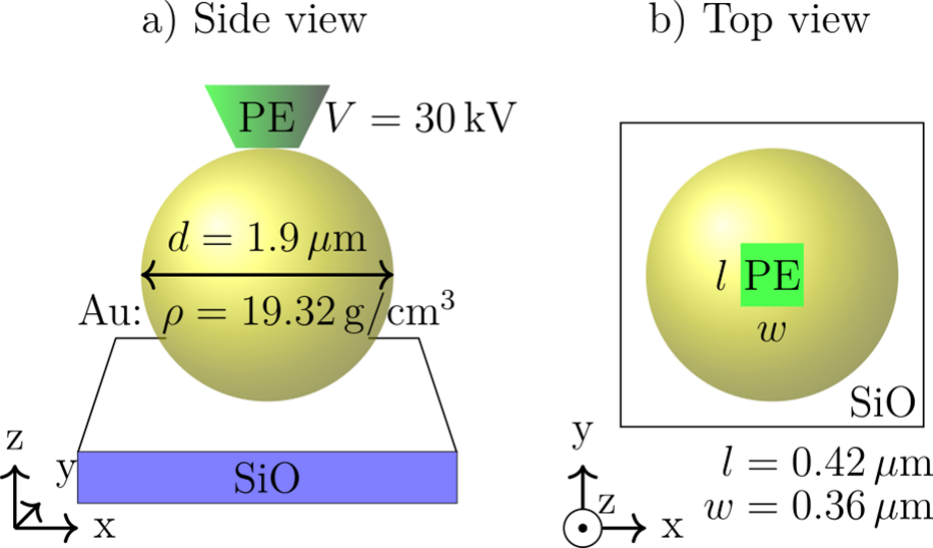
Input geometry, material, and beam parameters used in the Monte Carlo simulation. (a) Side view shows the impinging e-beam (green), the gold MP (yellow), and the thin SiO substrate (blue–white). (b) Top view displays the rectangular shape of the e-beam that corresponds to the scan field.

Figure 7a illustrates the geometry considered in the scattering simulation as a side view. The top view in (b) reveals the rectangular shape of the e-beam that can chosen such to agree with the scan field in the SEM. The depicted e-beam corresponds to a narrow scan region. The acceleration voltage of the PE is *V* = 30 kV and the amount of PEs is set to 1 · 10^7^*e* = 1.602 pC yielding sufficiently small statistical bounds to the obtained average values for energy loss, secondary electron yield, etc.

Two case studies were simulated to cover different magnification settings, i.e., scan field size with respect to MP size, as used during the experiments:

1. Narrow e-beam: The scan field has a length of *l* = 0.42 μm and width of *w* = 0.36 μm, which corresponds to a scan area that is smaller than the MP, as indicated by the green region in Figure 7b.
2. Broad e-beam: The scan field has a length and a width of *l* = *w* = 4 μm, which corresponds to a broad scan area compared to the MP as used for the experiment shown in Figure 2 for example.

The ratios of deposited charge *r*_C_ and energy *r*_E_ relative to the incoming number of electrons are shown in Table 2 for the narrow e-beam (middle column) and broad e-beam (right column). It is evident that both ratios are much higher for the narrow e-beam compared to the broad e-beam. In both cases, the deposited charge is negative, meaning that more electrons are deposited into the gold MP than emitted as SEs or transmitted electrons and BSEs. This is in good agreement with the theory presented in Figure 6 and the negative charging observed experimentally (see Section 2.3).

**Table 2:** Relative Charge, *r*_C_, and Energy, *r*_E_, Deposited in the MP for a Narrow and Broad E-beam Irradiating the MP, Respectively

property	1. narrow e-beam	2. broad e-beam
*r*_C_	31%	4%
*r*_E_	47%	7%

**Figure 8:**
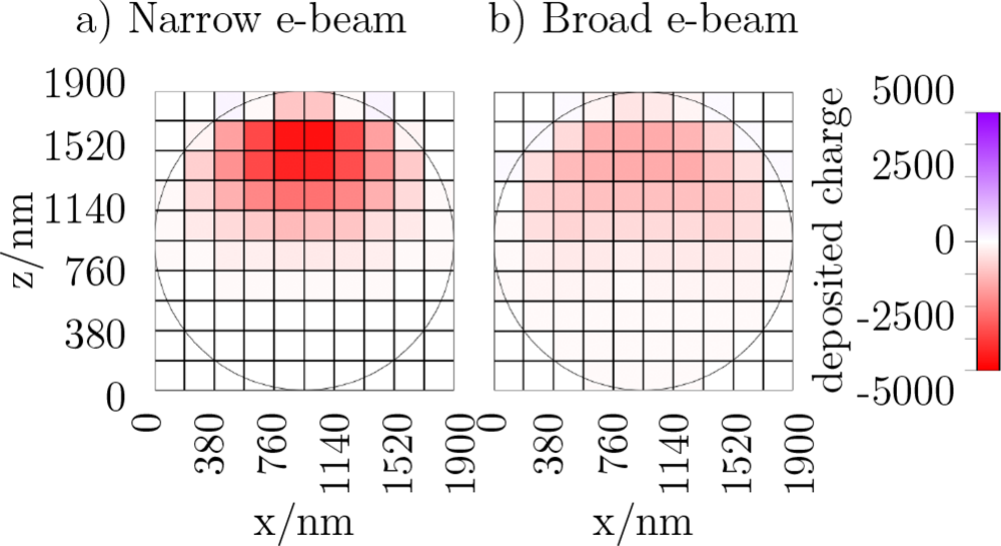
Simulated deposited charge distribution in the MP due to (a) narrow beam, as sketched in Figure 7, and (b) broad beam irradiation.

The slice cut along the *y*-direction, shown in Figure 8, represents a two-dimensional *x*–*z*-plane through the middle of the MP, which provide insight into the deposited charge distribution. The e-beam impinges the MP from the top. Figure 8a shows the spatial distribution for a narrow e-beam. The negative (red) deposited charge indicates the penetration volume of the electrons into the MP. The lower hemisphere, where the MP contacts the substrate, shows negligible deposited charge, reflecting that most of the electrons have been stopped in the upper part.

Figure 8b shows the distribution of the deposited charge for a broad e-beam. The absolute amount of the deposited charge is reduced compared to the narrow e-beam because the same amount of incoming electrons is distributed over a larger area, as also shown in Table 2. Again, the negative (red) deposited charge in the MP is mostly located in the upper hemisphere, where the e-beam enters the MP.

In the next sections, we further elaborate on the heating and charging introduced by the deposited energy and charge, respectively, considering also dissipation processes, which were not considered in the above scattering simulations.

#### 2.4.2 Thermodynamics

Heat is transferred to the MP as a result of a cascade of inelastic scattering processes between PEs and matter, e.g., starting with the excitation of a core electron, which subsequently decays into radiation and phonons, eventually involving intermediate excitation of electron–hole pairs, etc. In general, this is a transient process that involves a couple of subprocesses, which are partly unknown. For instance, the amount of secondary electrons ejected from the MP will change over time as the sample is charged in the electron beam (see Section 2.3). To describe the heating of the MPs, we will therefore focus on the most important processes, facilitating a semiquantitative description of the thermodynamics of the MPs under the electron beam.

The thermal energy (heat) *W*, which is transferred to and from the precursor MP, can be decomposed into different physical processes
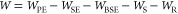
1where *W*_PE_ is the heat stemming from PEs; *W*_SE_ and *W*_BSE_, the heat dissipation through the SEs and BSEs leaving the gold MP; *W*_S_, the heat dissipation through the substrate; and *W*_R_, the heat dissipation through radiation (radiation losses).

When considering a narrow e-beam with a kinetic energy of *E* = 30 keV, a large fraction of PEs are stopped within the MP (see Section 2.4.1). Consequently, the e-beam transfers approximately *r*_E_ = 47% of its energy (which includes already the energy removed by BSEs and SEs) mostly in the form of heat to the MP. In the following we neglect the heat dissipation through the substrate because the area of the contact point of the MP with the substrate is of the order of nm^2^, which prevents an effective transfer of heat. Indeed, the contact point serves as a fuse, which eventually is responsible for burning a hole into the substrate (outcome HS) in reality. Radiation losses, however, constitute an important source of heat dissipation at elevated temperatures due to their *T*^4^ dependency (Stefan–Boltzmann law), which must not be neglected.

As a consequence, we obtain a transient heating of the gold MP, which starts with the electron beam hitting the MP, where it raises the temperature, which in turn increases radiation losses until the beam leaves the MP (during the scanning process) or the MP undergoes a phase transition, e.g., melts (outcome AG) or vaporizes (outcome NA). Neglecting for a moment the possibility of phase transitions, we approximately model this process of raising the temperature by equating the change of internal energy *U* (and hence temperature) of the MP with the net heat transferred to the MP
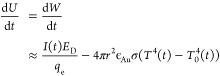
2where *I*(*t*) is the primary electron current on the MP, *E*_D_ the energy deposited by the electron beam, *q*_e_ the charge, *r* the radius of the MP, ϵ_Au_ the emissivity of the gold MP surface, σ the Stefan–Boltzmann constant, *T* the temperature of the MP, and *T*_0_ the temperature of the surroundings. The latter was introduced to take into account the heat transferred to the MP by radiation from the surroundings at room temperature, which may additionally change over time if the surroundings heats up. The deposited energy *E*_D_ is computed from the ratio of the scan area *A*_s_ used in the experiment and the beam area used in the Monte Carlo scattering simulation , the ratio of the deposited energy *r*_E_ = 0.07 in the broad beam setup (see Table 2), and the primary e-beam energy *E* = 30 kV according to
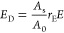
3The increase of the internal energy of the gold MP is linearly related to its temperature increase via
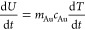
4with *m*_Au_ the mass of the particle and *c*_Au_ its specific heat capacitance. Combining the above equations, we end up with the following nonlinear first-order differential equation describing the evolution of the temperature of the gold MP
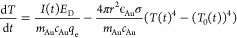
5

**Figure 9:**
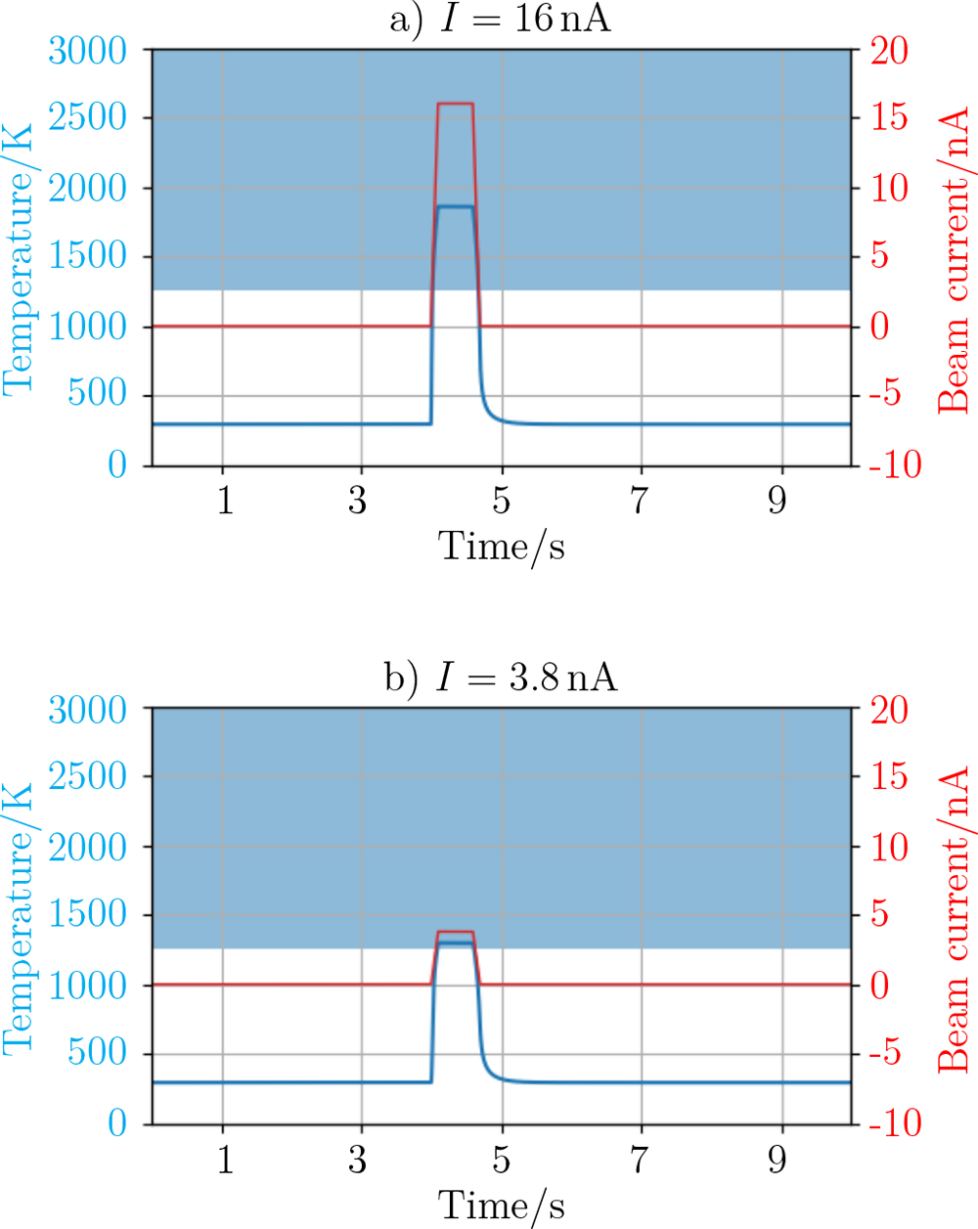
Time evolution of temperature within one frame of scan area *A* = 10 μm × 10 μm, employing *I* = 16 nA (a) and *I* = 3.8 nA (b) current. The blue shaded area indicates the temperature of the gas phase according to Figure 10.

We again note that this is only a first-order approximation, particularly neglecting inhomogeneous heating of the MP, internal and external convection currents, temperature- and size dependent material constants, etc. To solve the equation, we insert a time-dependent primary beam current of *I* = 16 nA (*I* = 3.8 nA), which sweeps in *t* = 0.68 s over the frame of *A*_s_ = 10 μm · 10 μm (see the red curve in Figure 9a,b, respectively). We furthermore assume an emissivity ϵ_Au_ = 0.7 that corresponds to an Au surface roughness of *R*_a_ ≈ 1 μm that is in the range of the curvature of the precursor Au MP.^39^ This is again a first order approximation, neglecting, e.g., the temperature dependence of the emissivity.

Under these assumptions, the calculated temperature (blue curve in Figure 9a,b) shows a strong increase to *T* ≈ 1875 K and *T* ≈ 1315 K, respectively, while scanning a frame and a very fast dissipation of heat and hence lowering of temperature within *t* = 1 s due to radiation when the beam is blanked.

In a more realistic approximation, the increase of the temperature is limited by first-order phase transitions. To take that into account, a calculation of a temperature–pressure phase diagram (CALPHAD method) of gold was performed by using Thermo-Calc^40^ and the SGTE Substance database.^41^

**Figure 10:**
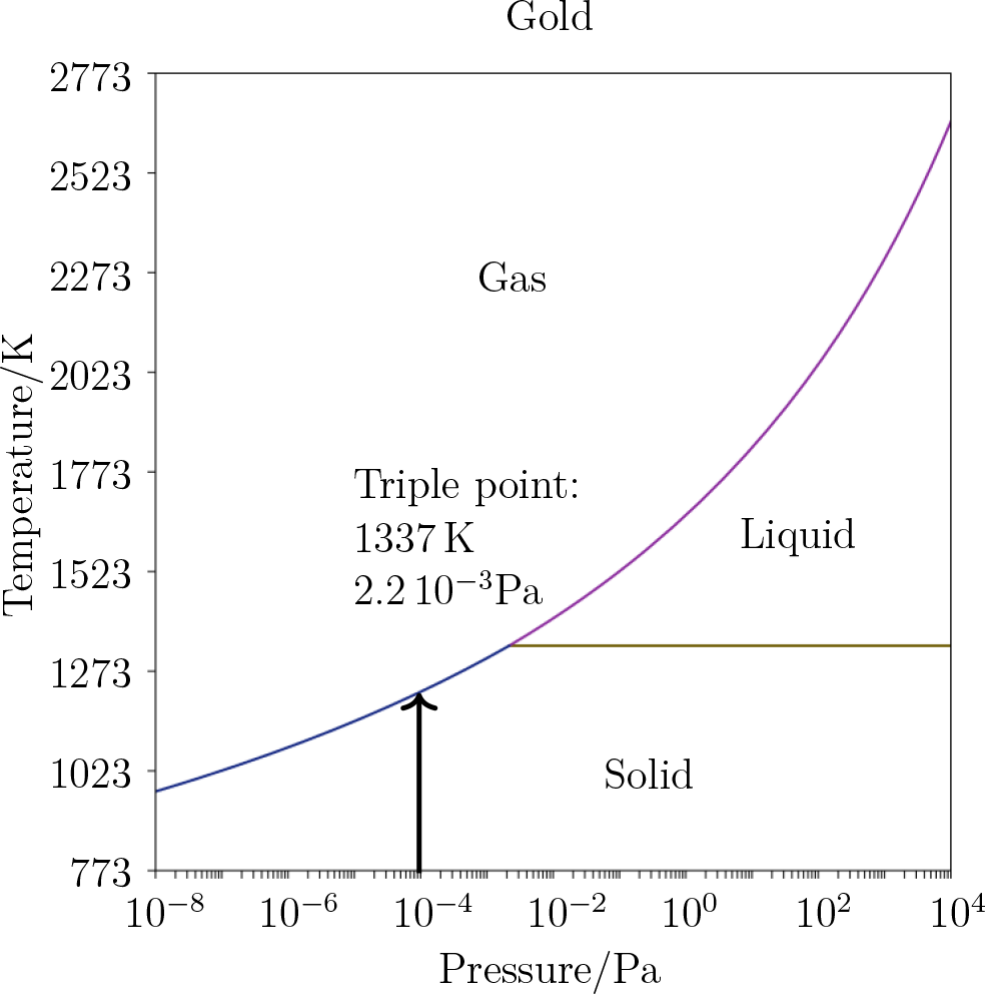
Calculated pressure–temperature phase diagram of gold. The black arrow denotes the pressure of *P* ≈ 3 × 10^–4^ Pa in the SEM chamber, where a phase transition appears at *T* = 1261 K.

Figure 10 shows the calculated *P*-*T*-diagram of gold. The triple point, at which gas, liquid and solid phases coexist, is found for gold at *P*_t_ = 2.2× 10^–3^ Pa and *T*_t_ = 1337 K. At lower pressure, only phase transition between solid and gas (sublimation) takes place, whereas at higher pressure, the transition between solid and liquid (melting) occurs. The environmental working pressure within the SEM chamber is *P* ≈ 3× 10^–4^ Pa (see the black arrow in Figure 10), 1 order of magnitude lower than the triple point. Hence, the bound gold atoms of the solid predominantly sublimate into the gas phase when the temperature exceeds *T* = 1261 K.

We therefore conclude that the temperature increase of the MP is considerably larger than the solid–gas phase transition temperature of Au in a high-vacuum environment (indicated by the shaded area in Figure 9) in case of *I* = 16 nA. That triggers a couple of complicated processes (vaporization of Au, Au atom convection within the MP, decomposing of the substrate), which are beyond our simple differential equation model. When inserting a current of *I* = 3.8 nA in the simulations, on the other hand, the temperature just reaches the phase transition temperature of *T* = 1261 K. Therefore, we can distinguish two limiting regimes that are observed experimentally:

- No phase transition takes place over the entire series of frames, i.e., after each temperature shock the Au MP returns to its original state. This regime is prevalent at reduced beam currents below some threshold, where the phase transition temperature is not reached. Here, the threshold of *I* ≈ 3.8 nA predicted by the simulations is somewhat lower than the experimentally observed one (*I* ≈ 5 nA, see Figure 4), which we attribute to the rather crude approximations employed in the simulations (e.g., neglection of temperature dependent parameters, heat diffusion through substrate, inhomogeneous heating).
- Thermal energy accumulation leads to an increase of the temperature beyond the phase transition temperature, triggering the sublimation of Au. At low external pressure the gaseous gold atoms travel along straight ballistic trajectories in vacuum, and eventually deposit on the substrate with a density decreasing with larger distance to the precursor. Furthermore, the substrate’s temperature is elevated by the deposited Au atoms as well as the radiation from the gold MP, allowing for the diffusion of gold atoms on the amorphous substrate and subsequent nucleation into gold NPs of various sizes (see also Figure 1).

#### 2.4.3 Charging

To elaborate the influence of charging similarly to the heat case, we decompose the charge *Q* transferred to and from the precursor MP into charging processes
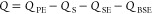
6with *Q*_PE_ the charge from the PEs, *Q*_S_ the electrons leaving the precursor MP through the substrate, *Q*_SE_ the secondary electrons, which have energies <50 eV, when they leave the matter, and *Q*_BSE_ the backscattered electrons, which have energies >50 eV.^32,34,36,42^

The MP precursor is negatively charged, as demonstrated in Figures 5 and 6, and further supported by Monte Carlo simulations (Figure 8). This suggests that there is a tendency for electron emission from the MP to restore equilibrium but no stimulus for charging-induced emission of positive gold ions as occasionally discussed in the context of e-beam irradiation with high-energy electrons.^24^

In contrast to thermodynamic modeling, we abstain from further modeling of the transient behavior of charging, because of a number of unknowns, introducing considerable errors in such a simulation. Notably, we do not know the resistivity of the MP substrate interface nor that of the substrate itself, preventing the modeling of e-beam-induced current (EBIC). Moreover, taking into account the transient charging and its impact on SE emission and SE recapture is very complicated.

## 3 Results and Discussion

### 3.1 Nanoparticle Synthesis

In the following we discuss the above results mainly focusing on their significance for the synthesis of NPs (other aspects like melting of MPs will be briefly discussed subsequently). The scattering simulations (Section 2.4.1) in combination with simple thermodynamic modeling (Section 2.4.2) offer a consistent explanation for the observed MP transformations, in particular the formation of NA (see Section 2.2). Consequently, we identify inelastic e-beam-matter interaction and the resulting heat deposited in this process as the driving physical mechanism for the observed behavior. Above a critical current of *I* = 5 nA, as shown experimentally in Figure 4, sublimation occurs (see Figure 10). The gaseous gold atoms partially redeposit on the substrate to form NPs (see Figure 1e–g) with sizes smaller than ≤400 nm.

The presented observations do not allow conclusions on the details of the NP formation process on the a-SiO_*x*_ substrate itself. We can therefore only speculate that the redeposition of gold atoms on the substrate is followed by additional diffusion, aggregation, radiation-induced collision, etc., eventually nucleating NPs, which then grow in size depending on the proliferation of Au atoms.^12^

The synthesized NA (see Figures 3c,d and 5) are stable over a long time. Furthermore, particle sizes of less than *d* < 10 nm are hardly accessible by chemical means. The smallest particle size found here is around *d* ≈ 3 nm.

To obtain further insight into the physical driving mechanisms of e-beam-induced fabrication of NPs at medium electron energy, we suggest a parameter study of the acceleration voltage of the PEs as well as the precursor MP size, thereby modifying the deposited energy and its balance with radiation losses and heat capacity. A further reduction of the complexity of the synthesis process, e.g., by using the e-beam in an SEM employing a fixed beam mode, would also be helpful.

The above results suggest that the fabrication of NPs composed of other (metallic) materials is possible under the following set of prerequisits: First, the phase diagram of the precursor material should show a solid-to-gas phase transition within the pressure regime of the SEM chamber.

Second, the solid-to-gas phase transition temperature must be attainable using low-to-medium e-beam acceleration voltages and moderate currents. Additionally, the shape of the precursor MP should exhibit a high surface-to-volume aspect ratio, which suppresses other heat dissipation mechanisms except the unavoidable radiation loss. The growth mechanisms on the substrate are also crucial factors that need to be considered in this process, although they fall outside the scope of this work.

Assemblies of metallic NPs are in turn promising candidates for tailoring the behavior of plasmons,^4^ photons,^43^ as well as acoustic surface waves.^44^ In particular, waves in disordered systems such as represented by the NA exhibit the tendency to localize, which in turn can be used for electromechanical gas and vapor sensors^45,46^ or flexible electrodes, e.g., for neural implants^47^ or monitoring of blood vessels.^48^

### 3.2 Microparticle Aggregation

The results of fabricating AGs using e-beam irradiation are outside the scope of this work; however, some findings remain noteworthy.

When multiple precursor MPs are irradiated with an e-beam, the local pressure in their vicinity increases due to gaseous gold atoms, potentially leading to a liquid phase near the surface of the MP. The resulting aggregated MP can appear elliptical or dome-shaped (as shown in Figure 3c,d), reflecting an incomplete liquid phase transition, which would otherwise yield a spherical shape due to high surface tension. By adjusting the irradiation dose and time, it is possible to tailor the growth of MPs to specific sizes, facilitating targeted fabrication for further application. Furthermore, combining this capability with micromanipulation techniques in an SEM enables the synthesized MP to be easily transferred to another location.

### 3.3 Hole in the Substrate

The fabrication of a hole in the substrate under the MP is influenced by electron conduction and thermal transport through the tiny contact area between the MP and substrate (see Figure 3d). By adjusting the irradiation dose and time, as well as the size of the MP, it is possible to inhibit this type of result. Furthermore, precise adjustment of all critical parameters enables the precursor MP to be used as a heating element at the microscale due to its tunable temperature, which can be controlled by varying the applied e-beam current. This property can be exploited for local modification of matter surrounding the gold MPs, e.g., synthesis of 2D materials.^49^

## 4 Conclusions

We developed a fabrication method to produce gold nanoparticles through low-energy electron irradiation of gold precursor microparticles deposited on a thin a-SiO_*x*_ substrate. Inelastic scattering of the electrons was identified as the primary physical mechanism driving the transformation of microparticles by beam-induced heating. Accordingly, the beam-induced heating can sublimate that precursor microparticles in vacuum if the e-beam current exceeds a threshold value that depends on the precursor shape, material, e-beam energy, and e-beam current. Subsequently, the deposition of the gaseous gold atoms on the surrounding substrate leads to nanoparticle nucleation and growth. This model allows predicting the feasibility of this nanoparticle synthesis route for other precursors, thereby guiding the synthesis of wide range of nanoparticles. Consequently, nanoparticle synthesis by e-beam irradiation opens up alternative avenues for nanotechnology relying on nanoparticles of various sizes and compositions that cannot be synthesized chemically. We particularly foresee applications in plasmonics and nanophotonics, among others.
